# Optimization and public transport tendering: a case study in Southern Italy

**DOI:** 10.1007/s12469-023-00324-9

**Published:** 2023-04-11

**Authors:** Vincenzo Corvello, Roberto Musmanno, Giuseppe Pavone, Francesco Santoro, Francesca Vocaturo

**Affiliations:** 1 Dipartimento di Ingegneria, Università di Messina, 98166 Messina, Italy; 2 Dipartimento di Ingegneria Meccanica, Energetica e Gestionale, Università della Calabria, 87036 Arcavacata di Rende, CS Italy; 3 Dipartimento Turismo, Marketing Territoriale e Mobilità, Regione Calabria, 88100 Catanzaro, Italy; 4 Itaca, 87036 Arcavacata di Rende, CS Italy; 5 Dipartimento di Economia, Statistica e Finanza “Giovanni Anania”, Università della Calabria, 87036 Arcavacata di Rende (CS), Italy

**Keywords:** Bus service, Transport cost, Graph partitioning, Districting, Mathematical programming, Adaptive large neighbourhood search

## Abstract

This article presents a multi-start heuristic approach to a design problem motivated by a real-world application in the Italian transport system. Specifically, it focuses on the problem of designing optimal lots in the public transport organization. In defining lots (in terms of number, size, and boundaries) both cost and service level have to be considered. Under certain assumptions, we model the problem as a graph partitioning problem and consider the same performance measure indicated by the relevant decree-law enacted by the Italian Ministry of Transport. The multi-start algorithm proposed for individuating high-quality solutions for the problem uses adaptive large neighbourhood search. The results of a computational study based on real data from a region in Southern Italy are reported.

## Introduction

The most important strategic decisions when planning train, subway, tram, ferry, or bus services are largely discussed in the scientific literature. From a practical perspective, in many territories (large cities, districts, regions, etc.) private companies operate public transport systems under concession contracts granted by the local governments (Sepúlveda and Galilea 2020). These contracts are generally used to set bilateral conditions between private operators and public authorities. In addition, they serve as an instrument to induce private operators in naturally non-competitive markets to act in accordance with social and environmental targets. In many countries, reform is taking place with the aim of changing public services gradually from production-oriented towards customer-oriented ones. Nowadays a reasonable level of access to mobility services is unanimously considered an essential right in a democratic society. Competitive tendering of concessions is frequently used to reach efficiency and effectiveness. In Sweden, e.g., more than 90% of the total public transport supply is tendered out through competitive contract-awarding procurement methods as recently emphasized by Jevinger and Persson (2019). Several studies in the scientific literature deal with tendering of concessions in the public transport sector. For instance, Mouwen and Rietveld (2013) focus on competitive tendering as a possible driving force for service quality enhancements and study the relationship between tendering and customer satisfaction. The authors present analytical results by referring to the situation in the Netherlands over the period 2001–2010. Cambini and Filippini (2003) analyse the main elements that a local authority must define in implementing competitive tendering procedures in the local public transport industry. In particular, they focus on the Italian regional bus sector which is characterized by the presence of economies of density and scale. Papaioannou et al. (2020) discuss European public transport tendering practices and analyse the reform in Cyprus.

We deal with public transport tendering from a different perspective by tackling a fundamental decision problem arising in this context, i.e., designing the service areas defined as *lots*. In particular, we consider the bus sector in Italy, but the results of our study can be easily extended to other transport modes and to other countries. In defining lots, in terms of size and boundaries, the decision-maker should consider both the service cost (efficiency) and the capacity to meet transport demand, with a special focus on customer satisfaction (effectiveness). In terms of efficiency, the size of the lots and the related quantity of transport services per year affect the unit cost of the service. The latter depends on the economy of scale and on the level of competition in tendering, which could decrease when the size of the lot increases, due to barriers to entry involving smaller operators. These two factors have a contrasting impact and, usually, for small-size lots the effect of the economy of scale is dominant; consequently, the unit cost initially decreases but, when this effect is reduced, the unit cost may increase due to the growth of barriers to entry on the market. In terms of effectiveness, the ideal solution consists of a single lot which coincides with the entire territory governed by the respective authority. In effect, this solution guarantees that the same operator serves any trip within the territory. It is worth stressing that journeys crossing the boundaries of lots may be served by different operators, with a consequent disutility for passengers; the related severity increases as much as the following elements decrease: the accuracy of planning and programming, the level of fare integration, the public capability in controlling the performance of concessions. In addition, the substantial overlap of several operators increases the risk of conflicts, especially in the event of changes of the services, due to changes of travel demand.

The decision problem just described can be formulated as a graph partitioning problem (Bichot and Siarry 2011) on an unweighted, connected, and undirected graph $G=(N,E)$. More specifically, it is a node-partitioning problem in which node set *N* regroups the municipalities, other administrative units considered indivisible, or specific hubs belonging to a region or basin (i.e., the territory of the respective authority for local public transport) and edge set *E* holds the streets. Note that only adequate streets can be considered in *E*, i.e., roads belonging to the local public transport network or, at least, roads potentially suitable for this purpose.

A general node-partitioning problem may be stated as follows. Let $\pi =\{L_1,L_2,...,L_m\}$ be a partition of set *N* into *m* subsets, i.e., $L_h \cap L_p = \emptyset \text{ for } h\ne p \text{ and } \bigcup _h L_h=N$. Partition $\pi$ is said to be feasible in *G* if each subgraph induced by $L_h$ ($h=1,...,m$) satisfies some constraints, depending on the specific application of interest. Let $\Pi (G,m)$ be the set of all feasible partitions. Given a function *f* defined on $\Pi (G,m)$, the decision problem becomes $min_\pi \{f(\pi ): \pi \in \Pi (G,m)\}$ whenever *f* represents an optimality measure to be minimized.

Another stream of literature related to our study concerns districting, i.e., the problem of grouping small geographic areas, called basic units, into larger clusters, called districts. The role of operations research techniques in districting problems keeps attracting much attention from the scientific community due to the broad scope of applications. In fact, these problems arise in various contexts ranging from political districting to sales districting and to school districting. The reader is referred to the chapter of Kalcsics and Ríos-Mercado (2019) and to the references therein for a recent overview on the topic. Three important criteria in districting are balance, contiguity, and compactness. Balance describes the desire for districts of equitable size with respect to some performance measure. Depending on the context, this criterion can either be economically motivated or have a demographic background. A district is called contiguous if it is possible to travel between the basic units of the district without having to leave the district [see Shirabe (2009) for details]. Finally, a district is said to be geographically compact if it is somewhat round-shaped, undistorted, and without holes.

Here contiguity is considered fundamental. In our node-partitioning problem it may result in the condition that each subgraph induced by a lot has to be connected. The aim is to find the set of lots that minimizes the sum of the total service cost. In addition, it is needed to control the maximum number of journeys crossing the boundaries of different lots in order to ensure an adequate service level. For the problem of interest, we propose a mathematical model with the sole aim of better describing it, especially its performance measure (objective function) and the service level constraint. Indeed, our main contribution is presenting an important real-life application in public transport and showing, through a case study in Southern Italy, the usefulness of operations research approaches to planning activities. For this purpose, we present a fast and effective heuristic algorithm based on adaptive large neighbourhood search (ALNS). The ALNS paradigm was introduced by Ropke and Pisinger (2006) and has been successfully applied to many decision problems in various real-world applications, e.g., small package shipping (Laganà et al. 2021), garbage collection (Laporte et al. 2010), simultaneous pickup and delivery (Hof and Schneider 2019), fleet deployment in the maritime environment (Bakkehaug et al. 2016), curriculum-based course timetabling (Kiefer et al. 2017), and the design of electronic circuits (Santos and de Carvalho 2018).

Mathematical models and optimization techniques have been extensively used in planning, operating, and controlling public transport systems. Most studies deal with service network design, real-time service controlling, vehicle and crew scheduling. For a recent literature review on this topic, with a special focus on bus service, the reader is referred to the article of Ibarra-Rojas et al. (2015). Recently Sheng and Meng (2020) have pointed out that the studies on public service contracting from an optimization perspective are relatively few. Specifically, these authors have analyzed the studies on public bus service contracting through awarding mechanisms (e.g., competitive tendering) and detect that optimization is rarely exploited. Surprisingly, studies from the “operations research/management science” category of WoS (*Web of Science*) account for a very low percentage of the total publications on the topic. From this perspective, the results presented in this article enrich the scientific literature, besides supporting policymakers in a fundamental administrative task.

The remainder of the article is organized as follows. Section 2 formally describes the problem of interest; specifically, Sect. 2.1 presents a mathematical model in order to better delineate the performance measure (i.e., the objective function) and the operational constraints; Sect. 2.2 provides details on the objective function by referring to the relevant decree-law enacted by the Italian Ministry of Transport. Section 3 presents the solution framework based on ALNS. Section 4 illustrates the outputs of an extensive computational study based on data concerning the Calabria Region in Southern Italy (case study). Conclusions follow in Sect. 5.

## Problem description

Computer-aided methods are attractive from an application perspective if data and criteria are clear enough to be amenable to mathematical representation. For this purpose, we formally describe the problem of interest and model it by using mathematical programming tools. We propose a synthetic version with the aim to better clarify the performance measure (objective function) and the way put in place to ensure the service level. Some constraints are just described into the model for the sake of simplicity.

Further notation is introduced here. First, we emphasize that decisions in public transport strongly depend on the behavior of the passengers who want to travel in the public transport network. Thus, integrating passenger data in public transport models is crucial (Schmidt and Schöbel 2015). In our model, let *P* be the total number of passengers in a given time period (usually an average weekday) in the area of interest. Specifically, *P* is determined by the number of people taking a bus trip which begins from and ends to a node in *N*. In more detail, for each node $i\in N$, $p_{ii}\ge 0$ represents the number of passengers that travel inside *i* in the fixed time period, whereas $p_{ij}\ge 0$ represents the number of passengers that move from node *i* to node $j\ne i$. We can set $P=\sum _{i\in N}\left( p_{ii}+\sum _{j\in N:j\ne i}p_{ij}\right)$.

Recall that we have to define the subsets that form a partition of *N*; these subsets are referred to as lots, coherently with the term used in public tenders in the (regional) bus service sector. Specifically, $L_h$ represents the *h*-th lot, whereas $H=\{1,2,...,m\}$ identifies the set of lot indices. Note that a lot can be empty, include a single node (*singleton*) or aggregate two or more elements of *N* (*non-singleton*). If *m* is set equal to |*N*|, then there is no limit on the number of non-empty lots that can be defined in practice (at most equal to the number of territorial units).

Moreover, let $c_i$ be the *transport supply* for node $i\in N$ whose measure is expressed in kilometers per year. This non-negative amount includes a part related to urban services (entirely internal to *i* and associated with internal bus trips) and a part related to interurban services (statistically associated with bus trips that begin from *i*). For the purposes of our decision problem, $c_i$ may be estimated by using linear regression models as described in Sect. 4.1.1 for the case study.

We can formulate our node-partitioning problem by defining the following groups of decision variables. Let $x_{ih}$ be a binary variable that takes value 1 if node *i* is assigned to lot $L_h$, and value 0 otherwise ($i\in N, h\in H$). For each *h*, the group of variables $x_{1h},...,x_{|N|h}$ defines a vector ${\mathcal {X}}_h$. Let $G({\mathcal {X}}_h)$ be the subgraph induced by the nodes assigned to $L_h$.

For the sake of clarity, we introduce further non-negative decision variables that depend on ${\mathcal {X}}_h$ (auxiliary variables). Specifically, let $C_h$ be the transport supply associated with lot $L_h$ ($h\in H$), i.e., the sum of the transport supplies concerning the nodes assigned to it; consequently, $C_h$ is expressed in kilometers per year. In addition, let $P^{ext}_h$ be the movement outwards associated with lot $L_h$ ($h\in H$); it is defined by the number of passengers moving from a node assigned to $L_h$ to a different node which does not belong to it. It is worth noting that journeys crossing boundaries of different lots may be managed by different operators, with related drawback/uneasiness for the passengers. From this perspective, we define threshold $\alpha$ ($0\le \alpha \le 1$) as the maximum percentage of passengers whose journeys can cross boundaries of different lots.

Finally, let *f* be a function which represents the total cost as defined in detail in Sect. 2.2. Here we just specify that *k* is a constant for our problem, whereas there is a part of cost which depends on the specific configuration (solution). In particular, given the transport supply $C_h$ associated with lot $L_h$, $g(C_h)$ returns a part of the unit standard cost for that lot.

### Mathematical model

A synthetic version of our graph partitioning model is given in the following.

1 $$\begin{aligned}{} & {} \text {Min} \quad \quad {f=k+\sum _{h\in H}g(C_h)C_h} \end{aligned}$$

2 $$\begin{aligned}{} & {} s.t.: \quad \quad \sum _{h\in H}x_{ih}= 1 \; \; \forall ~ i \in N \end{aligned}$$

3 $$\begin{aligned}{} & {} \quad \quad G({\mathcal {X}}_h) \text{ is } \text{ connected } \; \; \forall ~ h \in H: \sum _{i\in N}x_{ih}\ge 2 \end{aligned}$$

4 $$\begin{aligned}{} & {} \quad \quad P^{ext}_{h} = \sum _{i\in N}\left( \sum _{j\in N:j\ne i}p_{ij}x_{ih} - \sum _{j\in N:j\ne i}p_{ij}x_{ih}x_{jh}\right) \; \; \forall ~ h \in H \end{aligned}$$

5 $$\begin{aligned}{} & {} \quad \quad \sum _{h\in H} P^{ext}_{h} \le \alpha P \; \; \end{aligned}$$

6 $$\begin{aligned}{} & {} \quad \quad C_{h} = \sum _{i\in N}c_{i}x_{ih} \; \; \forall ~ h \in H \end{aligned}$$

7 $$\begin{aligned}{} & {} \quad \quad x_{ih} \in \left\{ 0, 1\right\} \; \; \forall ~ i \in N, \forall ~ h \in H \end{aligned}$$

Objective function (1) minimizes the total cost. Equations (2) represent assignment constraints; they ensure that each node is assigned exactly to one lot. Constraints (3) impose that, for each $h\in H$ associated with a non-empty and non-singleton lot $L_h$, the subgraph $G({\mathcal {X}}_h)$ induced by its nodes is connected. These constraints ensure contiguity/connectivity for two or more municipalities that compose the same lot. Equations (4) determine the total movement outwards (number of passengers going out) for each lot. Note that these equations can be written analogously as:

$$\begin{aligned} P^{ext}_{h} = \sum _{i\in N}\sum _{j\in N:j\ne i}p_{ij}x_{ih} (1- x_{jh} )\; \; \forall ~ h \in H. \end{aligned}$$

Inequality (5) limits the journeys crossing boundaries of different lots by ensuring that the movement outwards does not exceed a given percentage of the total movement (*service level constraint*). Equations (6) determine the transport supply associated with each lot (amount used in the objective function). Finally, constraints (7) guarantee that the components of ${\mathcal {X}}_h$ are binary, for each *h*. Auxiliary variables $P_{h}$ and $C_h$ ($h \in H$) will result in non-negative ones without imposing further constraints. Problem (1)−(7) is NP-hard and the solution of instances of practical interest is possible only via heuristic methods.

The literature proposes various linearization techniques for a non-linear model like the one described above. For instance, constraints (4) may be linearized by using an approach similar to the one presented in Fan and Pardalos (2010). In addition, there exist several ways to rewrite constraints (3) and ensure contiguity/connectivity by explicitly referring to the variables of the model. For instance, some authors resort to constraints similar to the subtour elimination inequalities used in routing problems to guarantee the connectivity of the routes [see, e.g., Ríos-Mercado and Fernández (2009); Salazar-Aguilar et al. (2011)]. Finally, we may discuss the symmetry that affects the proposed model and the opportunity of introducing the well-known symmetry breaking constraints. Anyway, dealing with this type of theoretical and methodological aspects is beyond the scope of this work. As mentioned above, the model has been introduced in this article with the aim of formalizing the performance measure and the service level constraint for our problem and facilitating the comprehension of these key components.

### Transport supply and standard cost: Italian practice

In general, the data concerning the number of people taking a bus trip are readily available at the respective authority for local public transport. The same does not apply to transport supplies that are expressed in kilometers per year. Usually, transport supplies are computed through statistical models (see Sect. 4.1.1 for details).

Here we deal with the standard cost whose importance in public transport is well explained by Petruccelli and Carleo (2017): “Knowing the standard cost of public transit services is essential both for contracting authorities to banish and competently perform public tender and for states or regions to allocate resources for public transport between local authorities according to the actual local needs”.

Given a lot associated with a transport supply ${\mathcal {C}}\ge 0$, its unit standard cost (Euros per kilometers) can be expressed as follows:

$$\begin{aligned} {\left\{ \begin{array}{ll} 1.46083\gamma + r(s) + g({\mathcal {C}}),~&~ \text {if}~{\mathcal {C}}>0 \\ 0,~~&~ \text {if}~{\mathcal {C}}=0, \end{array}\right. }\\ \end{aligned}$$

where $\gamma$ is a factor correlated to the transport system modernization, *s* represents the commercial speed (in kilometers per hour), *r* is a function of *s* and *g* is a function of ${\mathcal {C}}$. Our assumptions are based on a study by the Italian Ministry of Transport which was formalized through a national decree-law in 2018 (Italian Ministry of Transport 2018). In particular, the formula was obtained via a regression analysis by involving the most relevant factors that affect the transport cost: (*i*) size of the lot in terms of transport supply; (*ii*) commercial speed, and (*iii*) age and, as a consequence, depreciation of the vehicles.

The last factor affects $\gamma$. In our study, we assume that $\gamma$ is equal to 0.37 for urban services and 0.34 for interurban services. Note that the Ministry of Transport refers to these values as the average annual depreciation per kilometer for a bus which provides an urban service and an interurban service, respectively. Consequently, the first term of the unit standard cost is $1.46083\gamma =0.5405071$ for an urban service and $1.46083\gamma =0.4966822$ for an interurban service. According to the ministerial decree-law, function *r*(*s*) can be expressed as follows:

$$\begin{aligned} r(s)=-0.59230s+0.50837v_1(s-17)+ 0.06827v_2(s-32), \end{aligned}$$

where $v_1=0$ if $s\le 17$, $v_1=1$ if $s>17$, $v_2=0$ if $s\le 32$, and $v_2=1$ if $s>32$. It is reasonable to suppose that the commercial speed also depends on the typology of service. We hypothesize that $s=21.2$ km/h for urban services and $s=30.6$ km/h for interurban services (more information is given in Sect. 4.1.2). Then, we have

8 $$\begin{aligned} r(s)= {\left\{ \begin{array}{ll} -10.421606,~~ {\text {if}}\, s=21.2\, {\text{(urban services)}} \\ -11.210548,~~ {\text {if}}\, s=30.6\, {\text{(interurban services)}}. \end{array}\right. } \end{aligned}$$

Finally, according to the ministerial decree-law, our function $g({\mathcal {C}})$, which appears in objective function (1), can be expressed as follows:

$$\begin{aligned} g({\mathcal {C}})= {\left\{ \begin{array}{ll} 13.8927, &{} \text {if } {\mathcal {C}} \le 1 \text { (million kilometers),} \\ 14.07855 - 0.18583~ {\mathcal {C}}, &{} \text {if } 1< {\mathcal {C}} \le 4 \text { (million kilometers),} \\ 13.6656 + (0.0206~ {\mathcal {C}} - 0.16518)~ {\mathcal {C}}, &{} \text {if } 4 <{\mathcal {C}} \le 10 \text { (million kilometers),}\\ 14.07855, &{} \text {if } {\mathcal {C}}> 10 \text { (million kilometers).} \end{array}\right. } \end{aligned}$$

As mentioned above, the transport supply for node *i*, i.e. $c_i$, is composed by an urban part and an interurban part, and both parts are known (input data). Consequently, ${\mathcal {C}}$ also can be decomposed in an urban part denoted as ${\mathcal {C}}_u$ and an interurban part denoted as ${\mathcal {C}}_v$, i.e., ${\mathcal {C}}={\mathcal {C}}_u+{\mathcal {C}}_v$. Then, the total cost for the lot associated with ${\mathcal {C}}={\mathcal {C}}_u+{\mathcal {C}}_v$ becomes

$$\begin{aligned} (0.5405071-10.421606)~ {\mathcal {C}}_u +(0.4966822-11.210548)~ {\mathcal {C}}_v+g({\mathcal {C}})~{\mathcal {C}}, \end{aligned}$$

i.e.,

$$\begin{aligned} -9.8810989~ {\mathcal {C}}_u -10.7138658~ {\mathcal {C}}_v+g({\mathcal {C}})~{\mathcal {C}}. \end{aligned}$$

We observe that, in the objective function (1), the constant *k* can be obtained as

9 $$\begin{aligned} k=-9.8810989~ {\mathcal {C}}_u^{tot} -10.7138658~ {\mathcal {C}}_v^{tot}, \end{aligned}$$

where ${\mathcal {C}}_u^{tot}$ and ${\mathcal {C}}_v^{tot}$ represent the total urban transport supply and the total interurban transport supply, respectively (these quantities are known).

## A multi-start algorithm

In order to find a good solution to our problem in a reasonable time we propose a multi-start algorithm and denote as *maxit* the number of allowed iterations. For a comprehensive literature review on multi-start methods the reader is referred to the article of Martí et al. (2013). In each iteration, the algorithm builds an initial solution and applies ALNS with the aim of improving it. We recall that a solution is represented by a partition of the node set of *G* in subsets. Each subset induces on *G* a connected subgraph and represents a lot. During the search, $\alpha$-*infeasible* solutions for which the service level constraint (5) is violated can be generated. If a solution of this type arises, then a penalty, i.e., an additional term in objective function (1), is used in computing its total cost. In particular, the penalty is given by $\rho ({\overline{P}}-\alpha P)$, where ${\overline{P}}$ represents the movement outwards and coincides with the left-hand side of constraint (5) for the $\alpha$-infeasible solution, while $\rho$ represents a self-adjusting penalty coefficient. The pseudocode of our solution framework is reported in Algorithm 1, where $best_i$ represents the best feasible solution found during the *i*th iteration and *best* represents the best feasible solution found during the overall search.
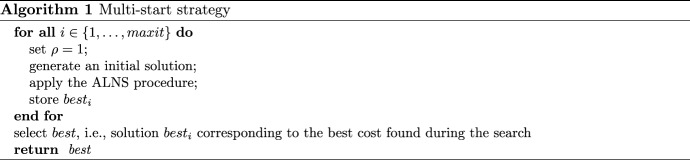


### Generation of initial solutions

The phase of generation of initial solutions aims at finding a given number of lots out of *G* that are built starting from a set of nodes called *seeds* whose selection strategy will be shown hereinafter. At this point we will have a number of lots each composed by a single seed node. Then, each non-seed node will be iteratively assigned to a lot. The initial phase can be split into two separate steps:

- Seeds selection. Let $F: |F| = \eta <|N|$ be the set of nodes with the highest value of transport supply. Practically, $s<\eta$ nodes are randomly picked from *F* and included in a set of seeds *S*. The procedure returns the starting lots each composed by a single seed.
- Nodes aggregation. Let $R=N\setminus S$ be the set of non-seed nodes. The procedure aims at assigning each unassigned non-seed node to a lot, as outlined in Algorithm 2. The “best lot for *r*” is the lot containing the node *n* such that the value $score = (p_{nr} + p_{rn})^{1/dist}$ is maximum, where *dist* is the distance between *r* and *n* by considering the adjacency matrix. If $p_{nr} + p_{rn} \le 1$ then $score = 1 / dist$.

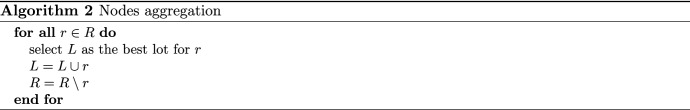


We can generate different initial solutions by changing $\eta$ and *s*; in addition, differences arise by carrying out more selections from *F* (with fixed $\eta$ and *s*).

### Improvement of initial solutions

The improvement phase is carried out through ALNS, i.e., a search paradigm based on a destruction/reconstruction principle. Once an initial solution is found, part of it is smashed by a destroy operator, while keeping the remaining part fixed. A new solution is then rebuilt by mending the destroyed part with a repair operator. This solution can be accepted or rejected by some acceptance criterion. The destruction/reconstruction steps are iterated until some termination criterion is met. We review the main elements of a generic ALNS framework and introduce specific features of our algorithm.

1. *Partial solutions and large neighborhood*. Let $N'\subset N$ be a subset of nodes of *G*. A partial solution is represented by a partition of $N'$ in subsets. Each subset induces on *G* a connected subgraph and still represents a lot. At each iteration at least $q=\lceil 0.1 \times |N|\rceil$ nodes are removed by using a destroy operator and reinserted, in a different way, by using a repair operator ($\lceil \ell \rceil$ denotes the smallest integer greater than or equal to $\ell$). Partial solutions arise during the destruction/reconstruction steps since some nodes are stored in a list ${\mathcal {L}}$ waiting to be reassigned to some lot. In particular, this list is gradually filled during the destruction phase and gradually emptied during the reconstruction phase. During the destruction/reconstruction steps, the number of lots can vary.
2. *Penalty updating*. When the ALNS procedure starts, the penalty coefficient $\rho$ is equal to 1. Every 10 iterations, it is multiplied by $2^{\frac{b}{10}}$, where *b* is the number of $\alpha$-infeasible solutions encountered in the last 10 solutions.
3. *Adaptive search*. The selection of destroy and repair operators is based on a roulette wheel selection mechanism. In other words, the destroy and repair operators are associated with specific weights. Given *t* operators with weights $w_i$, the *j*-th one is selected with probability ${w_j}/{\sum \limits _{i=1}^t w_i}$. The probabilities are computed by considering destroy operators and repair operators separately.
4. *Score and weight adjustments*. The search is divided into a number of segments defined as 50 iterations of the ALNS algorithm. Specifically, the weights are updated every 50 iterations by using the scores obtained during the last segment. In the first segment the weight of every operator is equal to 1, and at the start of a segment the score of every operator is equal to 0. The score of the selected pair of destroy and repair operators is increased by 30, 10, and 5 if their application results, respectively, in a new best feasible solution, in a (feasible or $\alpha$-infeasible) solution improving the current one, and in an accepted (feasible or $\alpha$-infeasible) solution not improving the current one. At the end of each segment, new weights are calculated by using the recorded scores. More specifically, let $w_{i,j}$, $\lambda _{i,j}$, and $\theta _{i,j}$ be the weight of the *i*-th operator in the *j*-th segment, the score of the *i*-th operator obtained during the *j*-th segment, and the number of times the *i*-th operator has been used during the *j*-th segment, respectively. If $\theta _{i,j}=0$, the algorithm sets $w_{i,j+1}=w_{i,j}$, otherwise it sets $w_{i,j+1}=\left( 0.9 \times w_{i,j}\right) +\left( 0.1 \times \frac{\lambda _{i,j}}{\theta _{i,j}}\right) .$
5. *Acceptance and stopping criteria*. Feasible or $\alpha$-infeasible solutions that are better than, equal to or slightly worse than the current solution could be accepted. In other words, a feasible or $\alpha$-infeasible solution is accepted if its objective value is less than the objective function value of the current solution multiplied by a user-defined factor $\delta \ge 1$. In order to accept fewer and fewer worsening solutions during the search, this factor gradually decreases until it becomes equal to 1. Its initial value is 1.03. Every 10 iterations $\delta$ is updated in the following way: $\delta =\text {max}\{1, 0.999 \times \delta \}$. The ALNS algorithm terminates whenever the best (feasible) solution has not changed for 400 consecutive iterations.

The pseudocode of the improvement phase is reported in Algorithm 3. For the sake of simplicity, updating related to $\rho$ in the objective function and to $\delta$ in the acceptance criterion (every 10 iterations) is omitted. In addition, the adjustment of scores and weights for the operators is not reported in Algorithm 3.
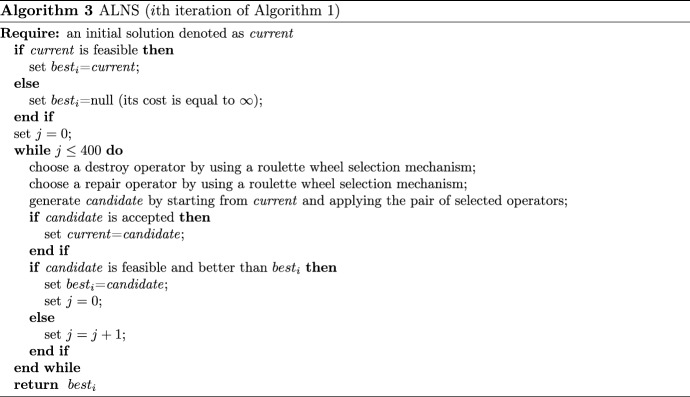


#### Destroy operators

Both partial and complete solutions have to be formed by lots that correspond to connected subgraphs of *G*. We illustrate the fundamental mechanism used in the destruction phase which is common to all operators.

**Fig. 1:**
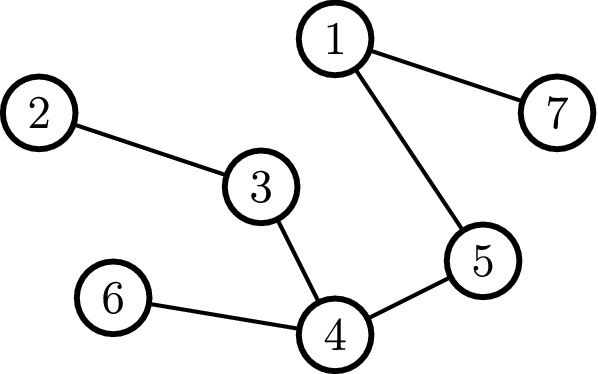
A representation of a lot

Consider the subgraph depicted in Fig. 1. Imagine that it corresponds to one among the lots that constitute a solution for our graph partitioning problem. Nodes 2, 6 and 7 can be removed without removing others since just one connected component remains. Instead, if the removal of a node implies the formation of two connected components, the one associated with the inferior number of nodes is completely removed (the choice is random if the number of nodes for the two components is equal). For instance, if node 3 is removed, then node 2 has to be also removed, as well as if node 1 is removed, then node 7 has to be also removed. Finally, if node 5 is removed, nodes 1 and 7 have to be also removed. Whenever more than two components remain after removing a node, the ones with a number of nodes less than $\lceil 0.03 \times |N|\rceil$ are taken out. Three different cases can arise: (*i*) just one connected component survives and will continue to represent the lot from which several nodes have been removed; (*ii*) no connected component survives, i.e., the lot disappears; (*iii*) $\varpi >1$ connected components survive; from that moment on, we will have $\varpi$ different lots at the place of the previous one. For instance, if $|N|=150$ and we remove node 4 from the lot depicted in Fig. 1, then the lot disappears (all nodes are taken out since $\lceil 0.03 \times |N|\rceil =5$ and the biggest component has three nodes). Therefore, a lot corresponding to a non-singleton set may disappear. Of course, a singleton set disappears if its unique node is removed.

Four operators are used to take out nodes and fill ${\mathcal {L}}$. These operators iteratively include in ${\mathcal {L}}$ a number of nodes greater than or equal to 1. This number is not predetermined and strongly depends on the selected node for the mechanism described above.

The first destroy operator is called *Random Removal* (RR). It iteratively selects nodes at random with the aim of diversifying the search. Specifically, at each iteration, a node *i* is randomly selected and removed. This operation can lead to the removal of other $\epsilon$ nodes, with $\epsilon \ge 0$. Then $\epsilon +1$ nodes are stored in ${\mathcal {L}}$; RR stops when the total number of nodes in ${\mathcal {L}}$ is greater than or equal to *q*.

The second destroy operator is called *Worst Service Removal* (WSR). It selects two nodes that appear to be placed in a wrong position in the current solution from a service perspective. Specifically, at each iteration, node *i* and node *j* belonging to different lots and corresponding to the maximum movement outwards are removed. In other words, the selected nodes are the ones associated with the maximum number of passengers moving between a pair of nodes belonging to two different lots. The removal of *i* and *j* can lead to the removal of other nodes from the respective lots according to the rules illustrated above (at least two nodes are stored in ${\mathcal {L}}$ at each iteration). The procedure stops when the total number of nodes stored in ${\mathcal {L}}$ is greater than or equal to *q*. Note that WSR tries to regain feasibility whenever constraint (5) is violated.

The third destroy operator is called *Worst Cost Removal* (WCR). It selects nodes that appear to be placed in a wrong position in the current solution from a cost perspective. Specifically, WCR preselects the nodes whose removal is linked to the removal of other $\lceil 0.01 \times |N|\rceil$ nodes at most. For each of them, WCR computes the saving (it considers the saving obtained by removing a group of nodes if the removal of a single node implies the removal of other nodes). The operator repeatedly chooses the node or the group of nodes associated with the largest saving until at least *q* nodes have been removed.

The fourth destroy operator is called *Connection Enhancement* (CE). It selects nodes that appear to be placed in a wrong position in the current solution from a connection perspective. Specifically, CE computes for each node *i* the difference $|A(i)|-a_i$, where $a_i$ is the number of nodes connected to *i* in the current solution, whereas *A*(*i*) represents the set of nodes adjacent to *i*; therefore, |*A*(*i*)| represents the number of potential connections. The operator repeatedly chooses the node associated with the largest difference and removes it. This operation can lead to the removal of other nodes according to the rules illustrated above. The procedure stops when the total number of nodes in ${\mathcal {L}}$ is greater than or equal to *q*.

#### Repair operators

Four operators are used to repair the current solution, i.e., reinserting all nodes stored in ${\mathcal {L}}$. We recall that only infeasibility with respect to the service level is allowed during the search. This consideration applies for both complete and partial solutions. Therefore, a node cannot be reallocated in a lot if it generates a disconnected subgraph.

The first repair operator is called *Greedy Construction* (GC). At each iteration, the operator extracts from ${\mathcal {L}}$ the node associated with the minimum insertion cost. It then inserts this node in the lot for which the minimum insertion cost has been computed. The process continues until all nodes have been inserted. Note that GC considers the insertion in all existing lots; in addition, for a node *i* corresponding to a large transport supply (i.e., $c_i$ greater than or equal to one million kilometers per year), GC also considers insertion in a new lot as singleton. It is worth specifying that a node may be non-allocable immediately since its adjacent nodes are also stored in ${\mathcal {L}}$. In this case, the insertion cost is set equal to infinity.

The second repair operator is called *Service Perspective Construction* (SPC). Differently from GC, SPC cannot generate new lots. This operator inserts the nodes one at a time by considering the service level. In particular, it preselects the nodes that are immediately allocable and, for each preselected node and for each lot, computes the sum of the passengers moving from it to the other nodes belonging to the lot. SPC chooses the node and the lot for which the maximum sum is reached. Then, it includes that node in that lot.

The third repair operator is called *Random Greedy Construction* (RGC). It represents a variant of GC since it introduces randomness in the first repair operator. The nodes corresponding to a transport supply greater than or equal to one million kilometers per year can generate new lots. At each iteration, RGC preselects the nodes that are immediately allocable in at least one existing lot or that can generate a new lot. Among these nodes, one is randomly chosen and included into a compatible lot for which the minimum insertion cost is achieved (the insertion in a new lot is evaluated only for the nodes corresponding to a large transport supply). We recall that a node and a lot are compatible if the node is adjacent to at least another node belonging to the lot.

The fourth repair operator is called *Balanced Construction* (BC). It represents a variant of SPC and cannot generate new lots. This operator considers the service level, but also balancing in terms of transport supply. At each iteration, BC selects the lot corresponding to the minimum transport supply compatible with at least a node in ${\mathcal {L}}$. Then, BC includes in the selected lot the compatible node corresponding to the maximum sum of the passengers moving from this node to the other ones of the lot.

## Computational experiments

Computational experiments were carried out on a PC equipped with a Intel core I9 9900K CPU running at 3.6 GHz, with 64 GB of memory. The multi-start algorithm based on ALNS was coded in *Java*. Data concerning the Calabria Region were used for the experimental phase.

### The case of the Calabria Region

The Calabria Region, with the Regional Law December 31, 2015, no. 35, and the approval of the Regional Transport Plan on December 19, 2016, redefined the cornerstones on the basis of which to redesign the entire local public transport system. The definition of the minimum service level, fares and strategic level programming of services took place between 2017 and 2019. On the basis of this programming, the regional government body responsible for the award of services by tendering, ART-Cal, in 2019 addressed the problem of defining the optimal tender lots, moving within the context of some general indications provided by the Region. A solution deriving from a commissioned study was submitted to the Italian Transport Regulatory Authority, ART, which issued its mandatory favourable advice in April 2020. Nevertheless, the procedure stopped (and was not yet restarted), due to the emergency deriving from the Covid-19 pandemic, during which significant changes in social, economic and transport systems took place.

More information on the case study is given in Sects. 4.1.1 and 4.1.2. Table 1 resumes the parameters and some values for the Calabria Region. All data used in the experiments are available from the authors upon request.

#### Transport supplies

Transport supply $c_i$ for each municipality $i\in N$ was computed by taking into account the methodological approach used by the Calabria Region. The estimate was made on the basis of linear regression models, where the independent variable is the quantity of services and the dependent variables are the population, the surface, the current amount of daily trips (by public transport) within the territory under consideration. More specifically, two linear regression models were used:

- the first one with the aim of determining the quantity of urban services to be programmed within each urban area (above a minimum population of 15,000);
- the second one with the aim of determining the quantity of interurban services to be programmed within each large area (by the institutionally adequate subject for it).

For the whole of Calabria, considered as the only large area, the application of the same methodology results in a quantity of services higher than that given by the sum of the individual areas, which translates into regional services, which reconnect the various areas, programmed directly from the Region. Since the quantity of services associated with each lot must include both the services totally internal to each lot and part of the services that connect lots among them, it is reasonable to be used as a dependent variable instead of the internal transport demand to the area, the transport demand originating in the area. On the other hand, for reasons of optimal organization of the service, the bus services between several lots are ordinarily attributed to the lot from which the transport request originates. The above estimate was referred to the minimum quantity of services, which was then adjusted in a mainly proportional manner to offer the maximum quantity of services possible with the available budget. Various aspects, including the optimal design of the lots, can lead to an optimization of costs and the possible use of savings to provide new services. Anyway, not including this estimate in the scope of this work, the quantity of necessary services can be taken as a fixed quantity associated with each node/municipality.

#### Other data

For each node $i\in N$, we must know $p_{ii}\ge 0$, which represents the number of passengers that travel inside *i* in the fixed time period and $p_{ij}\ge 0$, which represents the number of passengers that move from node *i* to node $j\ne i$. Recall that the matrix including these parameters has been used to control the number of trips that take place between different lots. For the Calabria Region, we can use the origin–destination matrix of the trips for work or study reasons produced by Istat (Italian Institute of Statistics) and referred to the resident population recorded at the 15th General Population Census (reference date: 9 October, 2011). It contains data on the number of people moving between municipalities, or within the same municipality, classified, as well as for the reason of the movement (work or study), by gender, means of transport, time slot of departure and duration of the journey. Any origin–destination matrix can be used for this purpose, but the choice of this matrix corresponding to trips with constrained destination seems to be a reasonable choice in the hypothesis of redefining the lots and the service, since the unconstrained trips (mainly for reasons other than work and study) could easily change as a result of the supply design. Note that for the Calabria Region we have a total movement equal to $P=77,683$ as indicated in Table 1.

In Sect. 2.2, the values reported for the commercial speed *s* have been computed by considering the case of the Calabria Region. These values have been utilized in the general formula (8) since we believe that they represent a good approximation of the commercial speed also for the other Italian Regions. In particular, the commercial speed for interurban services has been estimated on the basis of the average of the current speeds, whereas the one for urban services has been estimated on the basis of current public transport speeds and the average speed of vehicular traffic, establishing lower thresholds. Other data introduced in Sect. 2.2 and reported in Table 1 are ${\mathcal {C}}_u^{tot}$ and ${\mathcal {C}}_v^{tot}$, respectively, equal to 10,719,992 and 33,614,216 kms (per year) for the Calabria Region. In the objective function (1), the value of *k* can be obtained through Eq. (9) and is equal to $k=-466,063,500.2$ € for the case study.

**Table 1:** Parameters and some values for the Calabria Region

$$N=\{\text {Acquaformosa,Acquappesa,...,Zambrone,Zungri}\}$$		
$$|N|=409$$		
Description	Symbol	Total quantity
Urban transport supplies	$c_i^u$ ($\forall\ i\in N$)	${\mathcal {C}}_u^{tot}=\sum _{i\in N}c_i^u = 10,719,992$
Interurban transport supplies	$c_i^v$ ($\forall\ i\in N$)	${\mathcal {C}}_v^{tot}=\sum _{i\in N}c_i^v = 33,614,216$
Transport supplies	$c_i=c_i^u+c_i^v$ ($\forall\ i\in N$)	${\mathcal {C}}^{tot}=\sum _{i\in N}c_i = 44,334,208$
Number of passengers from *i* to *j* (inside *i* if $j=i$)	$p_{ij}\ (\forall\ i\in N, \forall\ j\in N)$	$P=\sum _{i\in N}\sum _{j\in N}p_{ij}=77,683$

### Numerical results and comparison

We run our algorithm by considering the case of the Calabria Region for which $|N|=409$. Specifically, in our algorithm we generated different initial solutions by considering different pairs $(\eta ,s)$, with $\eta$ varying between 12 and 15 and *s* varying between 5 and 10 (see Sect. 3.1 for their definition). In addition, for a same pair $(\eta ,s)$, several extractions from *F* were done, for a total of 216 initial solutions (216 also is the value of *maxit* in Algorithm 1). Note that we used the results of a preliminary experimental phase (tuning phase) to test different configurations and make decisions about operators and thresholds in the ALNS heuristic. Decisions about other components of Algorithm 1 have also been made in this phase.

Initially, we considered $\alpha =0.25$ to control the feasibility of the solutions according to constraint (5). We remember that it limits the journeys crossing boundaries of different lots by ensuring that the movement outwards does not exceed a given percentage of the total movement. This percentage value is considered a reasonable threshold at the Calabria Region. We obtained a best feasible solution (*best* in Algorithm 1) with an objective function value equal to 116,859,027.38 € and nine lots. In order to investigate the features of the algorithm, we point out that only eight initial solutions (out of 216) were $\alpha$-infeasible, whereas $best_i$ ($i=1,...,216$) at the end of ALNS (see Algorithm 3) is always feasible. In a single case the feasibility was retrieved after a long number of ALNS iterations (more precisely, 302). On average, the improvement in terms of solution quality ensured by ALNS was equal to 20.17%. The solution was obtained within a computational time equal to 7345 s (i.e., little more than two hours). This means that a single iteration of the multi-start algorithm required on average 34 s (an initial solution was built in approximately two seconds). All the operators in ALNS contributed to the improvement in terms of solution quality. However, RR seemed to be the best among the destroy operators, succeeded by CE (WCR and WSR were further away). The effect of RR is not surprising; many studies in the scientific literature confirm that randomness is fundamental in ALNS frameworks since it ensures a search diversification [see, e.g., Laganà et al. (2021)]. The contribution of the repair operators was more balanced; however, GC and RGC seemed to be slightly better than BC and SPC. In order to provide a measure of the improvement, we counted the number of times a new best solution was obtained. In addition, we summed the cost decreases. With respect to the number of times a new best solution was obtained by a destroy operator, we computed the following percentages by referring to the total number of improvements concerning all destroy operators: 55.95% for RR, 24.32% for CE, 11.50% for WCR, and 8.23% for WSR. Instead, with respect to the cost decreases, we computed the following percentages: 50.35% for RR, 34.27% for CE, 10.42% for WCR, and 4.96% for WSR. With respect to the number of times a new best solution was obtained by a repair operator, we computed the following percentages by referring to the total number of improvements concerning all repair operators: 30.73% for GC, 27.13% for RGC, 21.95% for BC, and 20.19% SPC. Instead, with respect to the cost decreases, we computed the following percentages: 28.74% for GC, 26.34% for RGC, 24.76% for BC, and 20.16% SPC. Among all destroy and repair operators, WSR seemed be the most ineffective. In anyway it was introduced prevalently to regain feasibility.

**Fig. 2:**
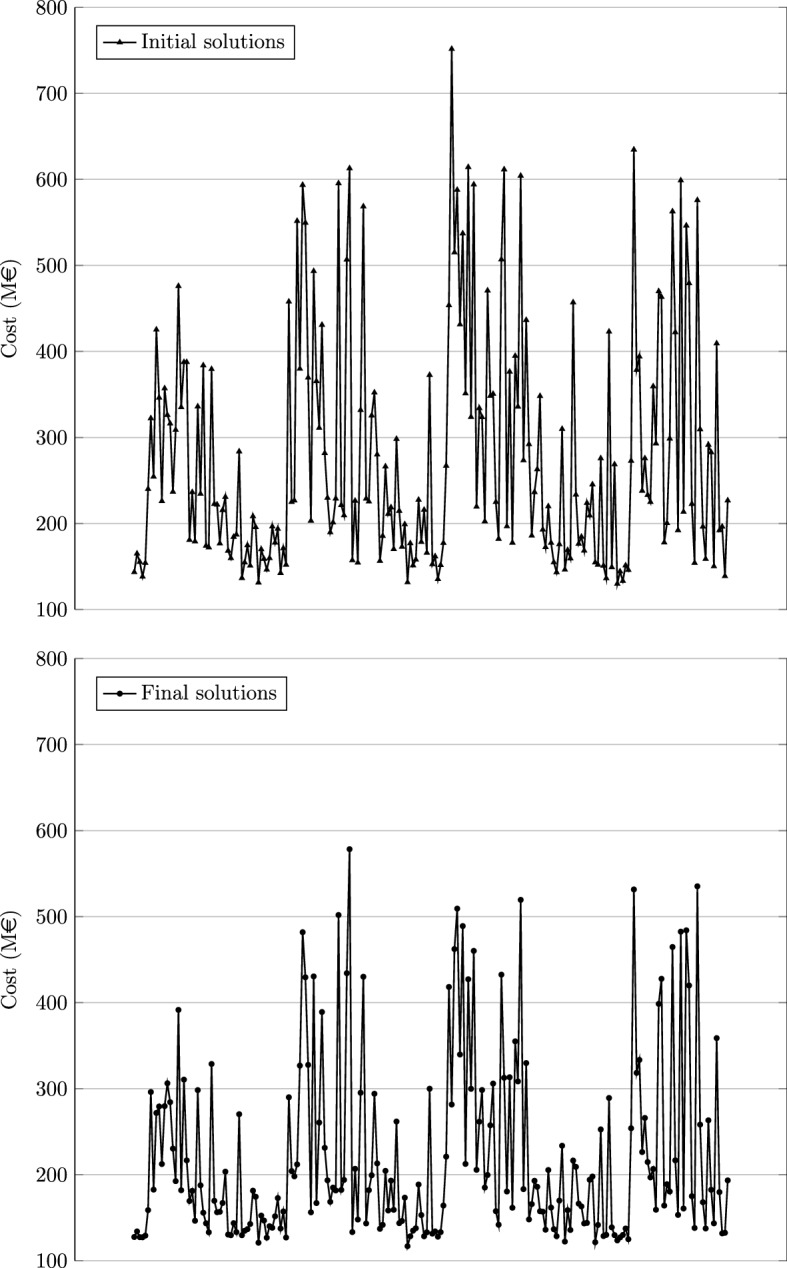
Differences in the cost values (in M€) for the various iterations of Algorithm 1

Note that $\alpha$ is important in this problem, since it affects the service level. Specifically, when the value of $\alpha$ decreases, the service level increases. In these cases, regaining feasibility represents a fundamental task since $\alpha$-infeasible solutions arise more frequently. In addition, note that the ALNS heuristic represents a fundamental component of our algorithm since it remarkably improves the initial solutions. In anyway, the generation of more initial solutions through the multi-start mechanism is even more important in this context. In fact, starting from different solutions ensures a good exploration of the search space (moving within the neighbourhood of a solution of low quality does not produce good results in general, even if the neighbourhood is large). Figure 2 shows the cost associated with the initial solutions (before applying ALNS) and final solutions (after applying ALNS) obtained within the iterations of Algorithm 1. For the sake of completeness, minimum, maximum and average values are also given in Table 2. The differences in the cost values are substantial for the various iterations of Algorithm 1. This aspect largely justifies the use of a multi-start scheme.

**Table 2:** Cost values

	Initial solutions	Final solutions
Minimum	129,922,596.95 €	116,859,027.38 €
Maximum	751,622,544.94 €	578,397,670.61 €
Average	277,182,645.10 €	221,265,499.46 €

**Table 3:** Main results

	$\alpha =0.20$	$\alpha =0.25$	$\alpha =0.30$
Objective function value for *best*	123,079,387.39 €	116,859,027.38 €	113,378,087.86 €
Number lots in *best*	10	9	10
ALNS average improvement	20.04%	20.17%	21.36%
Number of initial infeasible solutions	50	8	0

We ran again our algorithm by considering different values of $\alpha$. In particular, we considered 0.20 and 0.30 as an alternative to 0.25. Table 3 reports, for all alternatives, the main data specified for $\alpha =0.25$ above: objective function value for *best*, number of lots in *best*, average improvement obtained by ALNS, number of initial infeasible solutions. The computational results confirm that the cost increases when the service level increases. The average improvement ensured by ALNS is similar for the three values of $\alpha$. The computational times are also similar.

In order to assess the results obtained by our multi-start algorithm, we compared our best solutions with the four alternatives derived from a study commissioned by the Calabria Region to a pool of experts. These solutions, denoted as *sol*1, *sol*2, *sol*3, and *sol*4, include seven, seven, eight, and eight lots, respectively. We evaluated them by using the same data (adjacency matrix, origin–destination matrix of the trips produced by ISTAT, etc.) and the same objective function of the experiments described above. Table 4 summarizes the evaluation results. We point out that the objective function value does not vary with $\alpha$, since it coincides with (1) and does not include penalties. All solutions result $\alpha$-infeasible when $\alpha =0.20$. When $\alpha =0.25$, *sol*2 is feasible, but our *best* is significantly better than the former (improvement equal to 25.63%). Solutions *sol*1, *sol*2, *sol*3, and *sol*4 are all feasible when $\alpha =0.30$; in this case, the cost of the best competitor is 127,037,514.38 € corresponding to *sol*3. Our *best* dominates *sol*3; the improvement is smaller but still significant (10.75%).

We have not tested our model on an optimization commercial solver, since the size of the test problems that could be solved in this case would be very far from real cases and, consequently, the comparison with the corresponding heuristic solution would be of no practical interest.

**Table 4:** Competitors

	*sol*1	*sol*2	*sol*3	*sol*4
Objective function value	156,280,101.51 €	157,126,069.73 €	127,037,514.38 €	127,193,644.58 €
$\alpha =0.20$	$\alpha$-infeasible	$\alpha$-infeasible	$\alpha$-infeasible	$\alpha$-infeasible
$\alpha =0.25$	$\alpha$-infeasible	Feasible	$\alpha$-infeasible	$\alpha$-infeasible
$\alpha =0.30$	Feasible	Feasible	Feasible	Feasible

### Further experiments and managerial considerations

**Fig. 3:**
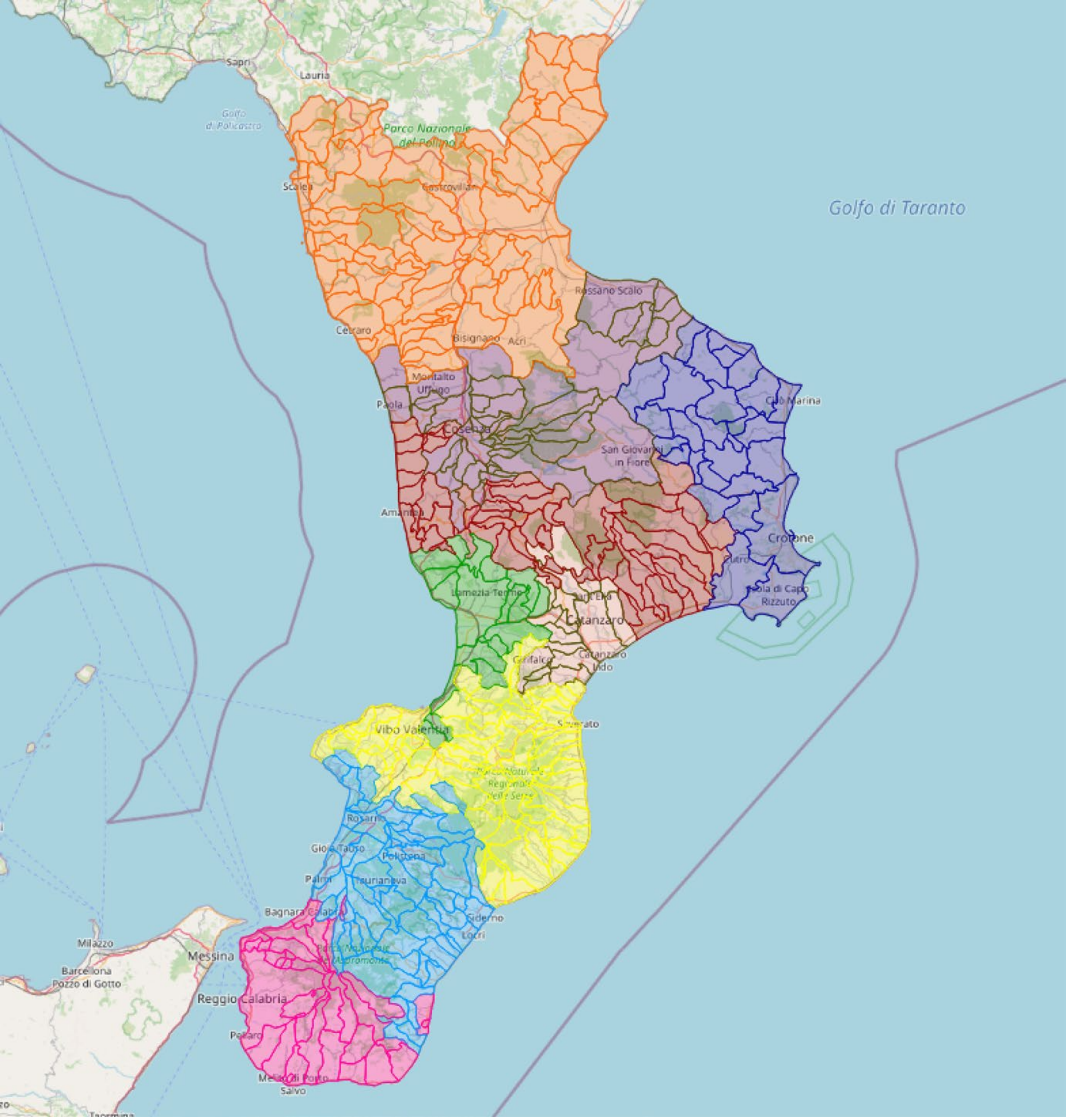
Pictorial representation of the best solution

We highlight that the numerical results obtained by our algorithm and reported above can be even improved by increasing computational time. Consider that designing optimal lots in the public transport organization represents a strategic activity. Therefore, in the case of a concrete application of our multi-start algorithm by public authorities, a very long time may be devoted to the solving process. From this perspective, we carried out further experiments by increasing the number of initial solutions, i.e., the number of iterations of Algorithm 1. The average time for a single iteration of Algorithm 1 recorded in all experiments is approximately equal to the value reported previously, i.e., 34 s. We also used other values of $\alpha$ in interval [0.2, 0.3]. The best solution found in this experimental phase by considering both the objective function and the service level has a cost of 111,794,041.14 €, nine lots, and a total movement outwards not exceeding 22% of the total movement (i.e., $\alpha =0.22$). The pictorial representation of this solution is reported in Fig. 3, where different colors are associated with different lots. Note that the pink lot seems to include a municipality isolated from the other municipalities included in the lot. Actually, this lot also amounts to a connected subgraph. This type of event can arise since there are few municipalities that correspond to indivisible administrative units but to disjoint territories. Our methodology may be easily adapted to different situations, if needed. For instance, we may: (*i*) eliminate the service level constraint 5 and use a weighted objective function where the total movement outwards represents a further cost; (*ii*) consider in the objective function only the service level to be maximized and introduce a budget constraint limiting the total cost; (*iii*) consider a fixed number of lots; (*iv*) limit the number of municipalities in a lot through a lower bound or an upper bound.

## Conclusions

The problem of designing optimal lots in the public transport organization is solved by the governments at the strategic level and is subject to very large investments. An effective decision support system in this context represents a key element in increasing efficiency and effectiveness of a fundamental public service.

In this work, the problem of designing optimal lots has been modeled as a graph partitioning problem and tackled by using a multi-start algorithm based on Adaptive Large Neighbourhood Search (ALNS), that is a local search approach in which simple operators compete to modify the current solution. The effectiveness of this algorithm has been empirically shown through computational experiments based on real data from the Calabria Region (in Southern Italy). We point out that our methodology can be easily adapted to other real cases and extended to other decision problems of interest for local governments, as detailed at the end of the section.

From a practical prospective, our results emphasize the need to develop technological instruments to support decision-makers in the public transport organization at the strategic planning stage, by investigating aspects of customer satisfaction. Recent studies in the scientific literature focus on passenger-oriented decision making in public transport at the tactical or operational level [see, e.g., the control measure, defined as stop-skipping, discussed by Gkiotsalitis and Cats (2021)].

The results we have obtained suggest the possibility of significant savings of financial resources, with respect to the solutions identified with non-algorithmic approaches. We strongly believe that our outcome will have a significant impact in the organization of public transport at least in Italy over the next few years. In this respect, consider that the annual financial support of the Italian government to all Regions is about 5 billion Euros. This amount does not take into account the extra budget allocated independently by each Region, which can vary significantly from case to case. As a result, in Italy, local public transport represents the second regional largest expenditure item, after healthcare.

Four main directions for future research can be identified. First, the model could be applied to other transport modes and to other contexts, besides the Italian bus transport sector, as far as the identification of lots is relevant. This includes other forms of public transport but also, for example, garbage collecting services. Second, the definition of lots could take into account social districts like Labor Market Areas (LMAs). In summary, LMAs represent geographical areas, precisely identified and simultaneously delimited throughout the national territory, where the citizens reside and work and where they indirectly tend to exercise most of their social and economic relationships [see, e.g., Casado-Díaz and Coombes (2011); Franconi et al. (2016)]. Third, while the objective of this paper was to apply operations research techniques to the problem at hand, proving their relevance and usefulness, future studies could focus on identifying solutions based on more efficient algorithms. In particular, different metaheuristics could be proposed in order to evaluate possible further improvements in the quality of the solution. This is the purpose of further future developments to be conducted also considering other test problems of the same type but on a larger regional scale. Uncertainty could also be addressed [see the review of De Maio et al. (2021) concerning other optimization problems in the transport area]. Fourth, as mentioned above, public transport strongly depends on the behavior of the passengers: the availability of a wider and more fine-grained database on the preferences of passengers could help to improve the overall model and, as a consequence, the decisions of the policymaker.
